# Supplementary material for: MicroRNA156 conditions auxin sensitivity to enable growth plasticity in response to environmental changes in Arabidopsis
Source: Nat Commun. 2023 Mar 22;14:1449. doi: 10.1038/s41467-023-36774-9 (PMC10033679; doi:10.1038/s41467-023-36774-9)
Supplement: Supplementary file 1 — Supplementary Information [file 41467_2023_36774_MOESM1_ESM.pdf]

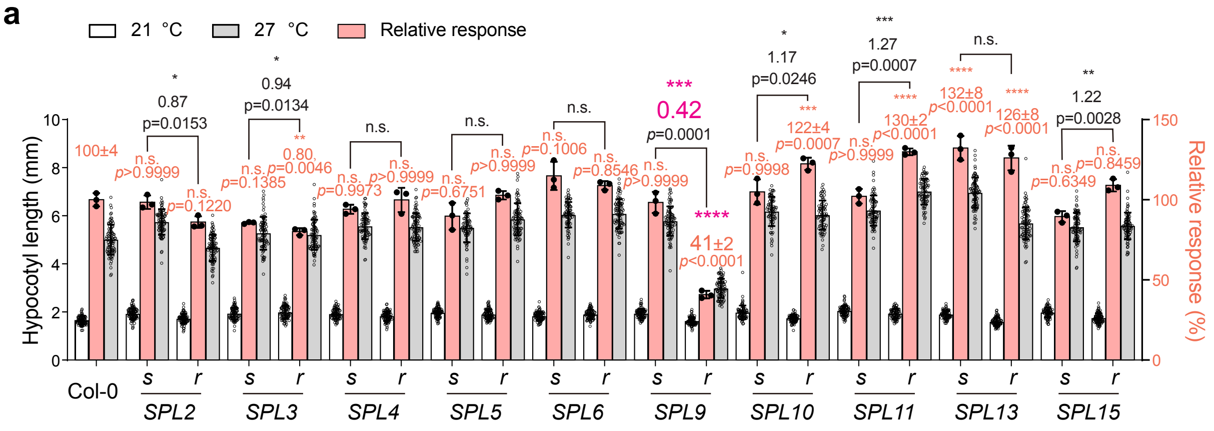

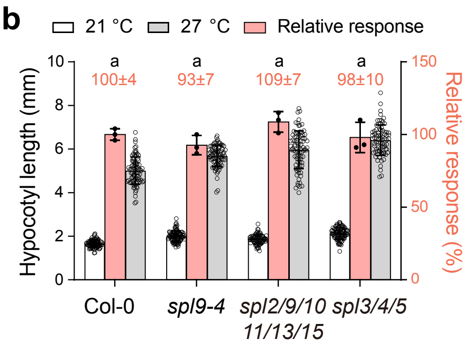

Supplementary Fig. 1. Thermomorphogenic hypocotyl growth is regulated primarily by SPL9. a Hypocotyl length measurements of 4-d-old seedlings expressing either miR156-sensitive (*s*) or -resistant (*r*) of *SPL2*, *SPL3*, *SPL4*, *SPL5*, *SPL6*, *SPL9*, *SPL10*, *SPL11*, *SPL13*, and *SPL15* grown under 50 μmol m^{-2} s^{-1} R light at 21 °C and 27 °C. The relative response to the warm temperature of each transgenic line was compared with that of Col-0 to identify the line(s) defective in the temperature response using one-way ANOVA (* $p < 0.05$, ** $p < 0.01$, *** $p < 0.001$, **** $p < 0.0001$), n = 3 independent biological replicates, and the *p* values are labeled in pink color. Among the 20 lines, *rSPL9* was the only line displaying a significant reduction in the hypocotyl response to warm temperatures. Also, the relative responses between the miR156 resistant and sensitive lines were compared to assess the role of each *SPL* in miR156-mediated thermomorphogenesis using a two-tailed Student's t-test (* $p < 0.05$, ** $p < 0.01$, *** $p < 0.001$), n = 3 independent biological replicates, the *p* values and fold changes are labeled in black color. n.s. indicates no significant difference. *SPL9* was the only gene displaying a more than twofold decrease in the relative response of the miR156-resistant (*rSPL9*) line compared with that of the miR156-sensitve (*sSPL9*) line. **b** Hypocotyl length measurements of 4-d-old Col-0, *spl9-4*, *spl2/9/10/11/13/15*, and *spl3/4/5* seedlings grown under 50 μmol m^{-2} s^{-1} R light at 21 °C and 27 °C. Pink numbers show the mean ± s.d. values of the relative responses and the lowercase letters indicate no significant difference in the relative responses (ANOVA, Tukey's HSD, $p < 0.01$, n = 3 independent biological replicates). For **a** and **b**, the open and grey bars represent hypocotyl length measurements at 21 °C and 27 °C, respectively. The pink bars show the relative response, which is defined as the relative hypocotyl response to 27 °C of a line compared with that of Col-0 (set at 100%). Error bars for the hypocotyl measurements represent s.d., n = at least 70 seedlings from three independent experiments; error bars for the relative responses represent s.d., n = 3 independent biological replicates. The centers of the error bars represent the mean. The source data underlying the hypocotyl measurements in **a** and **b** are provided in the Source Data file.

Supplementary Table 1. Primers for qRT-PCR analysis.

Accession	Gene name	Forward primer	Reverse primer
AT1G13320	*PP2A*	TATCGGATGACGATTCTTCGTGCAG	GCTTGGTCGACTATCGGAATGAGAG
AT2G43010	*PIF4*	AACCAGATCATCTCCGACCGGTTT	TCCCGCCGGTGAACTAAATCTCAA
