# Supplementary material for: MicroRNA156 conditions auxin sensitivity to enable growth plasticity in response to environmental changes in Arabidopsis
Source: Nat Commun. 2023 Mar 22;14:1449. doi: 10.1038/s41467-023-36774-9 (PMC10033679; doi:10.1038/s41467-023-36774-9)
Supplement: Supplementary file 3 — Description of Additional Supplementary Files [file 41467_2023_36774_MOESM3_ESM.pdf]

Description of Additional Supplementary Files

Supplementary Data 1. Abundance of annotated miRNAs in Col-0, *hyl1-2*, and *dcl1- 24/hyl1-2* at 21 °C and 27 °C. Worksheet 2 shows the abundance of annotated miRNAs in 4-dold Col-0*, hyl1-2*, and *dcl1-24/hyl1-2* grown under 50 µmol m^{-2} s^{-1} R light at either 21 °C or 27 °C. miRNAs were quantified as reads per million 45S rRNA reads. Worksheets 3-6 show the fold changes of annotated miRNAs between the two temperatures within the same genotype or between two genotypes at the indicated temperature.

Supplementary Data 2. Transcriptome analysis of Col-0, *pif457*, *MIM156*, *hyl1-2*, and *dcl1-24/hyl1-2* at 21 °C and 27 °C. Worksheet 2 shows the transcript levels (FPKM) of expressed genes in 4-d-old Col-0, pif457, MIM156, hyl1-2, and dcl1-24/hyl1-2 grown under 50 µmol m^{-2} s^{-1} R light at either 21 °C or 27 °C. Worksheets 3-6 show the fold changes of expressed genes between the two temperatures within the same genotype or between two genotypes at the indicated temperature.
